# Worldviews on Evidence-Based Cardiopulmonary Resuscitation Using a Novel Method

**DOI:** 10.3390/ijerph18189536

**Published:** 2021-09-10

**Authors:** Verónica V. Márquez-Hernández, Lorena Gutiérrez-Puertas, José M. Garrido-Molina, Alba García-Viola, Alfredo Alcayde-García, Gabriel Aguilera-Manrique

**Affiliations:** 1 Deparment of Nursing, Physiotherapy and Medicine, Faculty of Health Sciences, University of Almería, 04120 Almería, Spain; vmh380@ual.es (V.V.M.-H.); jgarrido22@gmail.com (J.M.G.-M.); albagarciaviola@hotmail.com (A.G.-V.); gaguiler@ual.es (G.A.-M.); 2 Research Group for Health Sciences CTS-451, Health Research Center, 04120 Almería, Spain; 3 Experimental and Applied Neuropsychology Research Group HUM-061, 04120 Almería, Spain; 4 Department of Engineering, University of Almería, 04120 Almería, Spain; aalcayde@ual.es

**Keywords:** cardiac arrest, cardiopulmonary resuscitation, database management, network analysis, worldwide research

## Abstract

The evaluation of scientific content by researchers, as well as the knowledge networks and working groups of cardiopulmonary resuscitation, can help to improve and expand new scientific evidence in this field. The aim of this study was to identify the global scientific publications on cardiopulmonary resuscitation research using a novel method. The method used was based on obtaining bibliographic data automatically from scientific publications through the use of the Scopus Database API Interface. A total of 17,917 results were obtained, with a total of 60,226 reports and 53,634 authors. Six categories were detected with 38.56% corresponding to cardiac arrest, 21.8% to cardiopulmonary resuscitation, 17.16% to life-support training and education, 12.45% to ethics and decision-making in cardiac arrest, 4.77% to therapeutic treatment, and 3.72% to life-support techniques. Analyzing and identifying the main scientific contributions to this field of study can make it possible to establish collaboration networks and propose new lines of research, as well as to unify criteria for action. Future research should delve into the analyses of the other elements involved in this area.

## 1. Introduction

Data from the World Health Organization (WHO) show that cardiovascular problems remain the leading cause of death worldwide [1]. Cardiovascular disease accounts for nearly 50% of all deaths, with a range of 30–65%, varying from country to country. Sudden death due to cardiac arrest is one of the greatest challenges that modern healthcare faces, not only due to the large number of cases involved but also due to its great social and economic impact [2]. In Europe, sudden cardiac arrest is the third leading cause of death [3].

Cardiac arrest can take place in the hospital or out of the hospital. The out-of-hospital cardiac arrest (OHCA) has a high incidence rate and low survival rate internationally [4]. However, the results can vary greatly between studies and regions. Specifically, it has been observed that the incidence rate of treated OHCA was higher in North America than in Europe, Asia, and Australia [5]. Regarding in-hospital cardiac arrest (IHCA), it is observed that of the total number of cardiac arrest cases that occur each year, up to 290,000 occur in the hospital setting. However, the outcomes of IHCA remain poor, with an average survival rate of less than 20% [6].

Although the guidelines for in-hospital and out-of-hospital cardiac arrest are almost identical, there are important differences between the conditions that must be considered. Significant variability in outcomes has been found in pre-hospital and in-hospital cases [7]. The technique of choice in both situations is cardiopulmonary resuscitation (CPR).

The CPR is a lifesaving technique that combines chest compressions and artificial ventilation. This technique has evolved over the last 50 years [8,9], and has caused an advance in research. The field of CPR continues to be dynamic with the emergence of new therapies and improvements in systems of care [10]. However, despite significant advances in the field of resuscitation science, important knowledge gaps persist [11].

The creation of the International Liaison Committee on Resuscitation (ILCOR) in 1992 has regularly brought together resuscitation experts from around the world to assess existing evidence, achieve a consensus on the science and, where appropriate, provide recommendations on CPR and emergency cardiovascular care [11]. The objective of this committee is to provide the resuscitation science community with a focused account of knowledge gaps identified during the evidence evaluation process.

This, in turn, has led to the creation and development of resuscitation guidelines such as those created by the European Resuscitation Council (ERC) [12], or the American Heart Association (AHA) [13]. The ERC has provided the standard for resuscitation guidelines and training in Europe and beyond [12]. In addition, AHA is dedicated to fighting heart disease and stroke [13]. Since 2000, researchers from the ILCOR member councils, including the two institutions mentioned above, have evaluated the latest scientific evidence on resuscitation in 5-year cycles [14]. This ultimately leads to resuscitation guidelines, such as The European Resuscitation Council Guidelines for Resuscitation 2021. The ERC guidelines provide guidance through its network of 33 national resuscitation councils. The guidelines are relevant for use in both the community (out-of-hospital) and hospital (in-hospital) settings.

In addition to these guidelines, the evaluation of scientific content by researchers, as well as the knowledge networks and working groups of CPR, can help to improve and expand new scientific evidence in this field. Furthermore, due to the development of Information and Communication Technologies (ICT), it is possible to access most of the scientific literature available through the internet [15,16]. However, this type of research does not take into account other aspects relevant to the scientific field, such as the structure of collaborative relationships between researchers [17]. The dynamism of these collaborations makes the analysis of the scientific collaboration network a topic of great interest [18,19]. This analysis enables us to answer these questions with an advanced method for analyzing scientific collaboration networks in universities and research institutions that has recently been developed [17]. Scientometrics and bibliometrics are developed to try to establish procedures and metrics for evaluating journal quality and the scientific output of researchers. Nevertheless, these methods do not take into account other aspects of interest [17].

The method developed by Montoya et al. [17] enables the extraction and use of features provided by the Scopus database to automate the search for manuscripts published by authors and institutions. Likewise, the information can be processed and analyzed at a later stage for different scientific purposes. The database chosen for the analysis in this method is Scopus, as it constitutes the largest database of peer-reviewed literature in different scientific fields [20]. This is a method of interest to researchers, research institutions and funding agencies [13], having already successfully published research using this tool [21,22]. The method opens new perspectives for the study of scientific collaboration networks because it can be applied at different levels of detail, from small research groups to large academic and research centers, and over different time frames [17].

However, regarding CPR, no investigations have been found in the existing literature that use this novel method to analyze scientific evidence published on this matter. Therefore, it is of particular interest to use this mathematical algorithm to understand the actual structure of global CPR research. The purpose of this study was to identify global scientific research on CPR research using a novel method.

## 2. Materials and Methods

### 2.1. Study Design

The method used was the use of software developed by Montoya et al. [17]. This method was based on obtaining bibliographic data automatically from scientific publications through the use of the Scopus Database Application Programming Interfaces (API) Interface. API is a communication interface that enabled access to an application’s data set. Subsequently, these data were analyzed using graphical visualization software and statistical tools [17].

### 2.2. Data Sources

The database used was Scopus, as it constituted the largest database of peer-reviewed literature in different scientific fields [16] and, compared to other large scientific databases such as Web of Sciences (WoS), it has a greater range of journals [23].

### 2.3. Search Strategy

Regarding the search strategy, the controlled vocabulary term “cardiopulmonary resuscitation” was used in the title, abstract and keywords in the Scopus database. The search was carried out in April 2020. The search strategy was (TITLE-ABS-KEY (Cardiopulmonary Resuscitation).

### 2.4. Procedure

**Figure 1:**
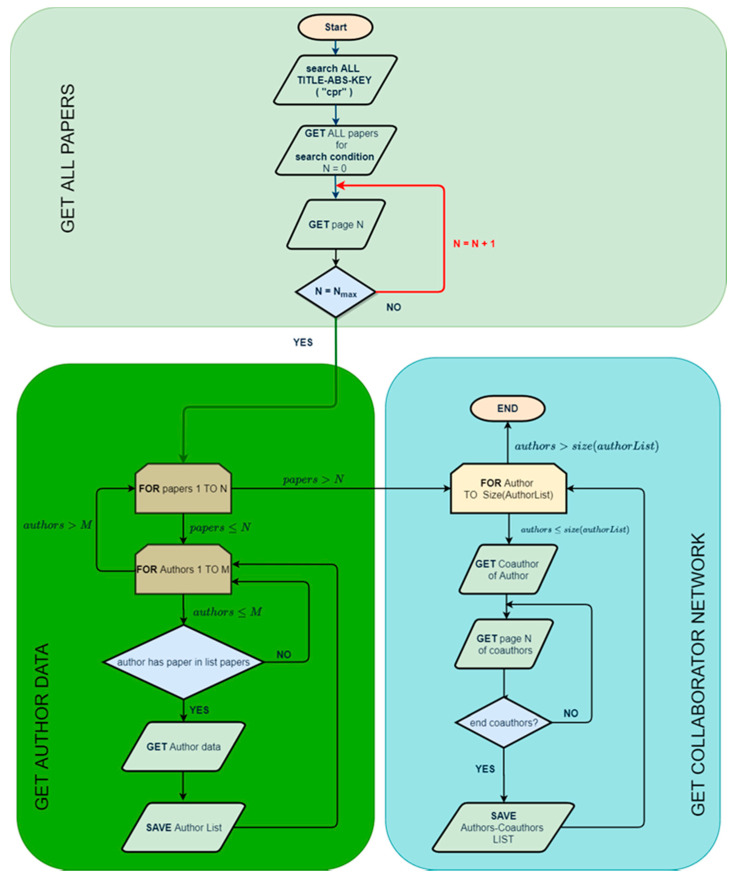
Data analysis procedure.

To perform the analysis and download the data, the bot, or web crawler, rasNetBot (https://doi.org/10.1016/j.tele.2017.10.010) (accessed 3 June 2021) was used. The web crawler was divided into three distinct blocks. In the first block the web crawler downloaded the information of all articles/papers that match the search criteria. In this case, a total of 17,917 articles were downloaded. Once downloaded, the program analyzed and obtained the list of authors and the list of papers with their respective keywords. The first list was used to feed the second block of the bot, which was in charge of downloading all of the author data. In this particular case, a total of 53,634 authors were downloaded. In the last program block, the various downloaded sources were navigated to obtain the relationships between them (Figure 1). The data extraction can be divided into the subsequent phases: (1) find article data (2) find author data and (3) find the collaboration networks.

The articles were represented by a node, with an arrow going through them to show the existing relationship between the two nodes, which was represented if article A cited article B.

## 3. Results

### 3.1. Characteristics of the Obtained Results

**Figure 2:**
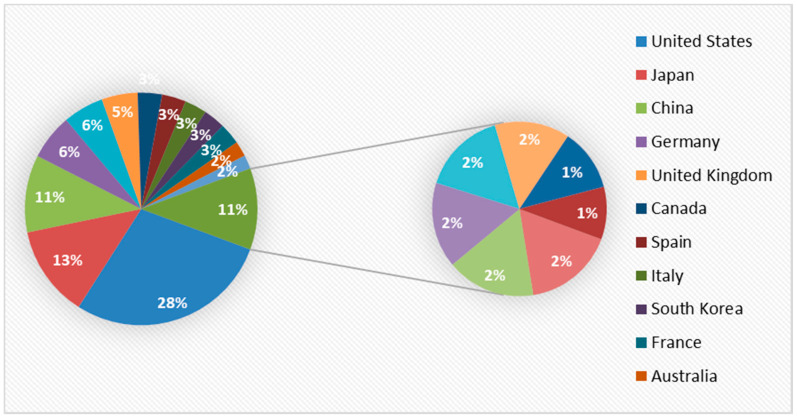
Origin of authors with studies on CPR.

**Figure 3:**
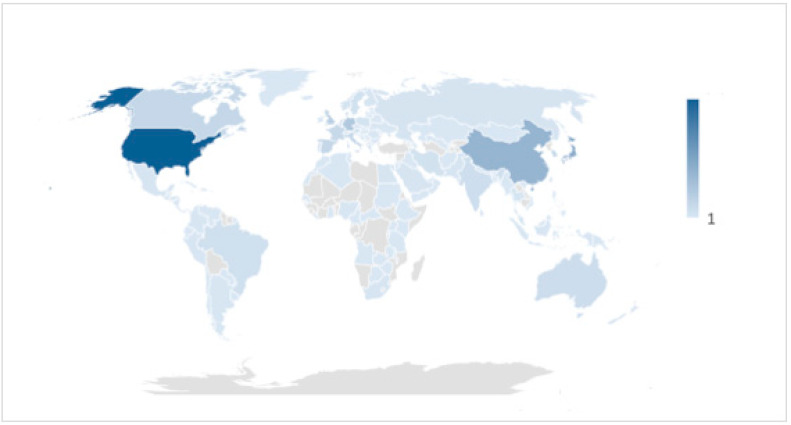
World map of CPR research.

A total of 17,917 results were obtained, with a total of 60,226 reports and 53,634 authors. Concerning the authors, the highest percentage, 28%, came from the United States, followed by 13% of authors from Japan. China was in third place, with 11% authorship, followed by Germany with 6%, and the United Kingdom with 5%. This information can be seen in detail in Figure 2 and Figure 3.

**Figure 4:**
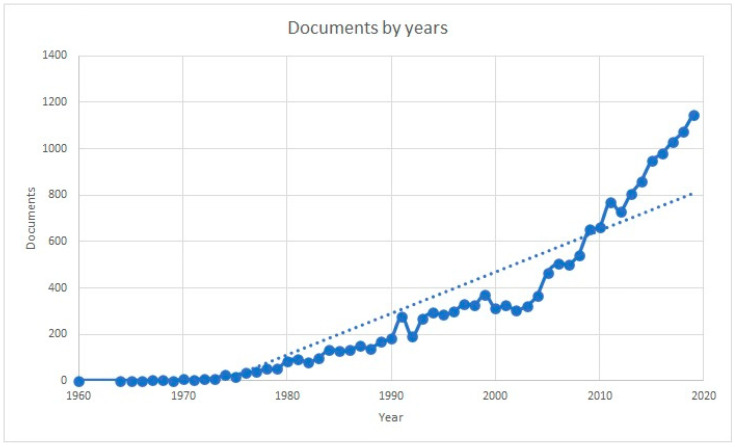
Annual production of CPR publications.

Regarding the annual evolution of publications, an analysis of the evolution was carried out from the year 1960. Evaluating the production using a polynomial equation, it was observed that production multiplied by 1.32 every 10 years (Figure 4). This highlighted the important current interest in research on CPR.

### 3.2. Categories Detected

**Figure 5:**
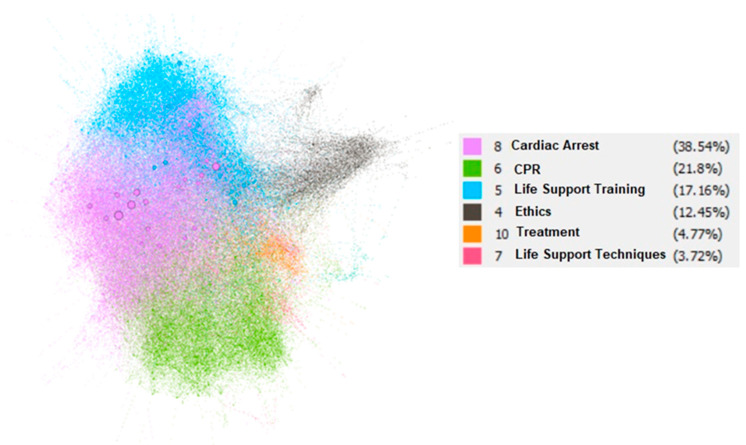
Representation of the 6 categories identified.

Six categories were detected, with 38.56% corresponding to cardiac arrest, 21.8% to CPR, 17.16% to life-support training and education, 12.45% to ethics and decision-making in cardiac arrest, 4.77% to therapeutic treatment, and 3.72% to life-support techniques (Figure 5). Each point in Figure 5 represented an investigation, establishing an edge between two points if there was a citation in both pieces of work. The color was assigned by a genetic algorithm that was detected based on how the nodes of a category were related. The thickness of each node was determined by the number of relationships with the other nodes.

Once the results were filtered, a genetic algorithm developed by the authors was carried out [17], which analyzed the extracted figure and grouped the different nodes by communities based on their relationships. The results of the two most prominent categories are described below. In each category, the most relevant results are grouped by subject.

#### 3.2.1. Cardiac Arrest Category

In this category, a total of 2395 investigations were found with 20,797 citations among them. Among the most relevant research within this category, the following aspects stood out: the importance of carrying out quality CPR following the guidelines and recommendations published on cardiac massage [7,24,25], early initiation [26,27,28], minimizing the interruptions that may arise during chest compressions [29,30,31], and handling other aspects of ventilatory support [32,33].

Firstly, CPR quality is a critical determinant of survival after cardiac arrest, suggesting the need for routine measurement, monitoring, and feedback systems during actual resuscitation [24]. The results of Abella et al. [25] suggest that highly trained health professionals often fall short of CPR guidelines during resuscitation efforts. There is considerable variation in the monitoring, implementation, and quality improvement. This ambiguity impedes the development of optimal systems of care to increase survival from cardiac arrest. The consensus statement of guidelines addresses the following key areas of CPR quality: metrics of CPR performance; monitoring, feedback, and integration of the patient’s response to CPR; team-level logistics to ensure the performance of high-quality CPR; and the continuous quality improvement of provider, team, and systems levels [7].

Secondly, it has been shown that the early initiation of CPR improves survival in a cardiac arrest. Specifically, dispatcher-assisted bystander CPR seems to increase survival in cardiac arrest [26]. The efforts to improve survival should focus on the prompt delivery of interventions by witnesses [27]. The preferable approach to resuscitation for adult patients who witnessed out-of-hospital cardiac arrest was cardiac-only resuscitation by bystanders [28].

Another important aspect of this category is minimizing the pauses that may arise during chest compressions. Wik et al. [29] found that chest compressions were not delivered at half the required speed, and most compressions were too shallow. In addition, Kern et al. [30] observed that mouth-to-mouth ventilation performed by single layperson rescuers produced substantial interruptions in chest-compression-supported circulation. Interruptions of precordial compression for rhythm analyses that exceeded 15 s before each shock compromised the outcome of CPR and increased the severity of post-resuscitation myocardial dysfunction [31].

Regarding ventilatory support, the outcome after CPR with chest compressions alone was similar to that after chest compression with mouth-to-mouth ventilation [32]. However, in out-of-hospital CPR, it was found that professional rescuers were observed to excessively ventilate patients [33].

To verify all of the above, feedback played an important role through new technologies [34,35]. Automatic feedback improved CPR quality [34] and could serve as a useful adjunct for rescuers during resuscitation efforts [35].

The number of investigations that indicated the importance of early defibrillation is prevalent [36,37,38,39,40]. Wik et al. [38] found that the patients with ventricular fibrillation and ambulance response intervals of longer than 5 min had better outcomes with CPR before defibrillation was attempted. Additionally, it was observed that the routine provision of approximately 90 s of CPR prior to the use of the automatic external defibrillator was associated with an increased survival when response intervals were 4 min or longer [39]. Finally, the quality of CPR prior to defibrillation directly affected clinical outcomes [40]. Training for health professionals in this field was key [41], as well as mass training for the rest of the population in order to reduce the incidence of deaths [42].

#### 3.2.2. CPR Category

In the CPR category, 1660 studies were found with a total of 9120 citations. The most important keywords were cardiopulmonary resuscitation with 11.06% of the total, cardiac arrest with 8.04%, followed by CPR with 2.46%, epinephrine with 2.20% and resuscitation with 2.11% (Table 1).

In this category, various relevant investigations were found in this field. Prominent amongst them were protocols and recommendations of the AHA, aiming to unify the criteria of CPR clinical practice [43,44]. The AHA provided a comprehensive review of evidence-based recommendations for resuscitation and emergency cardiovascular care [45]. On the other hand, The National Conference on CPR and Emergency Cardiac Care (ECC) developed standards and guidelines for CPR and ECC.

Several research studied the administration of epinephrine and its action on cerebral and myocardial blood flow [45,46]. These studies showed that a critical level of myocardial blood flow was required to restore the ability of the heart to function as a pump after prolonged CPR, and that a drug that increased blood flow improved resuscitation efforts [46].

The importance of performing quality CPR was highlighted again in this category, [47] as well as the indicators that enabled the verification of its effectiveness [48]. In addition, other aspects were addressed such as the effects from CPR maneuvers at the physiological level [49], the usefulness of capnography [50], the use of mechanical chest compression devices at the hemodynamic level [51], and the argument of the limited scientific evidence for the use of drugs in advanced life support (AVS) in the out-of-hospital context [52]. This study indicated that a shorter drug delivery time in animal models of cardiac arrest may be one reason for the failure of animal studies to translate successfully into the clinical arena [52].

## 4. Discussion

The objective of this investigation was to identify global scientific research on CPR research. First of all, the significant number of investigations found should be noted: 17,917 results. Nonetheless, this figure is far different from the number of results found in other investigations using the same system, such as the one carried out on malaria where 85,370 articles were found [22].

Secondly, considering the origin of research carried out on the subject, it was observed that the highest percentage of publications came from American authors. In the United States, cardiac arrest is one of the leading causes of death, with more than 356,000 out-of-hospital cardiac arrests annually, of which nearly 90% are fatal [13]. Thus, CPR is a major topic of scientific research. The second country identified was Japan, where death from cardiac-related issues has now become one of the main causes of death [53]. Acting in the event of cardiac arrest continues to be a global challenge [2], where various factors are involved. In this study, two main elements stood out: cardiac arrest and CPR.

Cardiac arrest was the main research area found in this investigation. Within this category, the research by Wik et al. [29] is prevalent, where the quality of CPR is analyzed during OHCA. OHCA continues to be a major global issue today [27], although in places like Japan recent research shows that survival rates after OHCA are gradually improving [54]. The second most relevant article in this category was written by Sasson et al. [27] where, much like the previous investigation, OHCA was of great relevance. In 2010, the authors carried out a systematic review and meta-analysis to explore the predictors of survival from OHCA and to emphasize the importance of bystander action. Therefore, the performance and quality of CPR in an OHCA situation constituted one of the main research topics within this category.

The second most prominent aspect in the results found in this investigation was CPR. In this category, the most noteworthy investigation was carried out by Michael et al. in 1984 [45]. This research analyzed the mechanisms by which epinephrine increases cerebral and myocardial perfusion during CPR in dogs. It should be noted that the article with the highest degree of importance within the category was not carried out in humans. The next relevant study was carried out by Rudikoff et al. [49], where the mechanisms of blood flow during CPR were explored. Unlike the first category, where the investigations focused mainly on the out-of-hospital context, the studies in this category were based largely on mechanisms and hospital treatments. Regarding this last aspect, the results of the IHCA literature indicate that efforts to prevent IHCA require both a system to identify deteriorating patients and an adequate interventional response [55]. The optimization of resources for patients with IHCA depends on the ability to provide the highest quality of care supported by the greatest evidence available [6]. Therefore, tools such as the one indicated in this study allow for the optimization of search resources and can help to find the best evidence on IHCA.

The rest of the categories found were training and education in life-support, ethics and decision-making in cardiac arrest, therapeutic treatment, and life-support techniques. These elements formed part of the international recommendations on CPR [12,13]. The 2015 ERC recommendations included a detailed discussion of the ethical principles that underpinned CPR. In addition, it highlighted the importance of training for both laypeople and health professionals and included the principles of resuscitation training [12]. With regard to this aspect, the importance of using feedback systems to improve education and training of rescuers was prevalent. Research, such as that carried out by Cortegiani et al. [56], highlighted the importance of training lay rescuers using feedback systems. Specifically, the authors obtained satisfactory results in chest compression training based on real-time feedback software. There were different results on the best approach for CPR performed by laypeople. Training guided by instructors with practice and feedback, as well as retraining, appeared to obtain favorable results in the CPR training of adult laypeople [57]. Nevertheless, it was necessary to further analyze these elements to obtain a global vision of the research on these aspects at the international level.

The method used is a novel tool that enables the evaluation of relationships between researchers, a group of researchers and organizations. However, the results of this study should be considered in the context of several limitations. Due to the abundant number of investigations detected, the main limitation was the in-depth analysis needed to extract all of the information found. As a result, it was necessary to summarize and highlight only the two most important elements for this investigation, as it was not possible to include all figures and graphics made. Nevertheless, future research will continue to carefully analyze the most important findings from the rest of the categories. In addition, the current health situation of the COVID-19 pandemic must be considered, thus, future research should consider using this tool when searching for results that enable the optimization and improvement of CPR care.

## 5. Conclusions

Cardiac arrest continues to be one of the leading causes of death worldwide, and proper action is essential to increase the survival of victims. For this reason, the terms cardiac arrest and CPR constitute the two main elements in the research found on the subject. Regarding cardiac arrest, the research focuses mainly on the out-of-hospital context whereas for CPR, the most important investigations focus on hospital mechanisms and treatment. This study highlights the importance of CPR research, which allows for evidence-based practice. The evaluation of scientific content by researchers, as well as the knowledge networks and working groups on cardiopulmonary resuscitation, can help to improve and expand new scientific evidence in this field. Analyzing and identifying the main scientific contributions to this field of study can make it possible to establish collaboration networks and propose new lines of research, as well as to unify criteria for action. Furthermore, being able to analyze the most researched aspects of the subject, the terms most used in research, and those topics that are less covered in CPR can help new researchers to focus their work on aspects that are lacking in current CPR research. This study contributes to the literature by generating new knowledge and proposing new lines of research related to CPR. Investigations such as this one allow for a better understanding of the variability in CPR research, as well as a better processing of the most relevant findings. Future research should delve into the analysis of the other elements involved in this area.

## Figures and Tables

**Table 1:** Most important keywords in the CPR category.

Keys	No. of Appearances	% of Total
Cardiopulmonary Resuscitation	624	11.06%
Cardiac Arrest	454	8.04%
CPR	139	2.46%
Epinephrine	124	2.20%
Resuscitation	119	2.11%
Ventricular Fibrillation	100	1.77%
Heart Arrest	63	1.12%
Hypothermia	58	1.03%
Vasopressin	54	0.96%
Coronary Perfusion Pressure	49	0.87%
Return of Spontaneous Circulation	33	0.58%
Asphyxia	31	0.55%
Outcome	29	0.51%
Cerebral Blood Flow	27	0.48%
Defibrillation	27	0.48%
Impedance Threshold Device	27	0.48%
Apoptosis	26	0.46%
Hemodynamics	26	0.46%
Active Compression–Decompression	19	0.34%
Blood Flow	19	0.34%

## Data Availability

All experimental data to support the findings of this study are available upon request from the corresponding author.
